# The dynamics of motor learning through the formation of internal models

**DOI:** 10.1371/journal.pcbi.1007118

**Published:** 2019-12-20

**Authors:** Camilla Pierella, Maura Casadio, Ferdinando A. Mussa-Ivaldi, Sara A. Solla

**Affiliations:** 1 Center for Neuroprosthetics and Institute of Bioengineering, School of Engineering, École Polytechnique Fédérale de Lausanne (EPFL), Geneva, Switzerland; 2 Department of Informatics, Bioengineering, Robotics and Systems Engineering, University of Genoa, Genoa, Italy; 3 Department of Physiology, Northwestern University, Chicago, Illinois, United States of America; 4 Shirley Ryan Ability Lab, Chicago, Illinois, United States of America; 5 Department of Physical Medicine and Rehabilitation, Northwestern University, Evanston, Illinois, United States of America; 6 Department of Biomedical Engineering, Northwestern University, Evanston, Illinois, United States of America; 7 Department of Physics and Astronomy, Northwestern University, Evanston, Illinois, United States of America; Johns Hopkins University, UNITED STATES

## Abstract

A medical student learning to perform a laparoscopic procedure or a recently paralyzed user of a powered wheelchair must learn to operate machinery via interfaces that translate their actions into commands for an external device. Since the user’s actions are selected from a number of alternatives that would result in the same effect in the control space of the external device, learning to use such interfaces involves dealing with redundancy. Subjects need to learn an externally chosen many-to-one map that transforms their actions into device commands. Mathematically, we describe this type of learning as a deterministic dynamical process, whose state is the evolving forward and inverse internal models of the interface. The forward model predicts the outcomes of actions, while the inverse model generates actions designed to attain desired outcomes. Both the mathematical analysis of the proposed model of learning dynamics and the learning performance observed in a group of subjects demonstrate a first-order exponential convergence of the learning process toward a particular state that depends only on the initial state of the inverse and forward models and on the sequence of targets supplied to the users. Noise is not only present but necessary for the convergence of learning through the minimization of the difference between actual and predicted outcomes.

## Author summary

Several studies have suggested that as we learn a new skill our brain forms representations, or “internal models”, of both the skill and the environment in which we operate. Theories of motor learning postulate that the brain builds forward models that predict the sensory consequences of motor commands, and inverse models that generate successful commands from movement goals. We test this hypothesis by taking advantage of an interface that relates the user’s actions to the position of a cursor on a computer monitor, thus allowing users to control an external device through body movements. We recorded the motions of the body and of the cursor, and used this data to estimate forward and inverse models. We followed the time evolution of these estimated models as interface users practiced and acquired a new skill. We found that the description of learning as a simple deterministic process driven by the presented sequence of targets is sufficient to capture the observed convergence to a single solution of the inverse model among an infinite variety of possibilities. This work is relevant to the study of fundamental learning mechanisms as well as to the design of intelligent interfaces for people with paralysis.

## Introduction

A distinct feature of the neuromotor system is the large number of muscles and degrees of freedom that allow it to attain a specific motor goal in a number of different ways [1]. This is both a resource and a computational challenge: while this motor redundancy provides the brain with a multitude of options, an enabling feature of motor dexterity, it also results in a family of ill-posed problems characterized by a lack of uniqueness in their solutions [2, 3]. Here, we consider the challenge posed by redundancy from the perspective of learning. How does the central nervous system learn to perform a novel task when multiple alternative solutions are available? This question acquires clinical relevance when a person suffering from loss of limb or some form of paralysis must reorganize the still available mobility to recover quality of life and independence through the operation of assistive devices–such as wheelchairs or robotic assistants–and dedicated human-machine interfaces. Are some possible solutions more easily learned than others? Could learning be facilitated by adapting the interface to the solution that the subject seems to be acquiring?

In the last two decades, studies of motor learning [4–8] have established that the adaptation of limb movements to external perturbing forces takes place through the gradual formation of an internal representation, or "internal model" of these forces. To be predictable, the forces cannot be random disturbances, but must have a deterministic structure expressed in relation to the motion of the body and to the brain’s commands [6–9]. Donchin et al. [10] and others [11–13] have proposed to represent the development of such an internal model as the evolution of a dynamical system.

Internal models are of two types: forward and inverse. Forward models owe their name to their predictive representation of the process that transforms action commands into their sensory consequences. Inverse models reverse the direction of this process by deriving action commands from desired sensory outcomes. Earlier theoretical work by Jordan and Rumelhart [14] considered how the learning of actions can be viewed as the concurrent learning of forward and inverse models of actions. They introduced the concept of distal learning, where the learner has to find a mapping from desired outcomes to actions in order to achieve a desired outcome. To do so, the subject begins by forming a predictive forward model of the transformation from actions to distal outcomes. Such transformations are often not known a priori, thus the forward model must generally be learned by exploring the outcomes associated with particular choices of action. Once the forward model has been at least partially learned, it can be used to guide the learning of an inverse model that predicts the action needed to achieve the distal outcome.

Here, we extend the distal learning approach to the learning of a novel map established by a body machine interface (BoMI) that translates movements of the upper body (shoulders and arm) into movements of an external object that users must guide to a set of target locations. This BoMI has been shown to be an assistive tool for people that have lost the use of their hands after injury to the cervical spinal cord [15–19]. However, the field still lacks a mathematical description of the process that takes place while subjects are learning to proficiently use this BoMI. An analysis of if and how people form an internal model of the interface, how this representation evolves with time and depends upon the initial state, will allow us to characterize the efficiency of the interaction with assistive interfaces, both for healthy subjects and for subjects with different types and levels of disability. This fundamental knowledge will facilitate the development of an advanced coadaptive interface, capable of handling changing motor abilities as well as changing operational demands. For this purpose, we investigate how unimpaired subjects become skilled at controlling an external object via the BoMI. Our findings are consistent with the hypothesis that learning proceeds through the concurrent evolution of coupled forward and inverse models of the body-to-object mapping established by the BoMI. The validity of this description is tested by comparing the evolution of motor performance predicted by the model with the actual learning performance observed in a group of human subjects. In addition, we compare the forward and inverse models derived from simulated learning dynamics with forward and inverse models estimated from motion data at different stages of learning.

## Results

### A model of learning while practicing control via a body-machine interface

We investigated how users of a body machine interface learn to reorganize or "remap" their body motions as they practice controlling an external object through the BoMI. The controlled object could be a wheelchair, a robotic assistant, or a drone [16, 17, 20]. Here we focus on the control of a computer cursor whose two-dimensional coordinates determine its location on a computer screen. Effectiveness in cursor control is the first and most common benchmark for interfaces based on neural activity [21–23], as the ability to control two-dimensional position is readily applied to a variety of tasks (e.g., an action performed via a joystick, entering computer text, etc.). We consider interfaces in which a linear mapping associates the body motion signals to the coordinates of the external object. Importantly, there is an imbalance between the dimensionality of the task space and that of the body signals, the latter being larger. Thus, any position of the controlled object corresponds to many, potentially infinite, different body configuration signals.

The BoMI matrix *H* establishes a linear map between these two spaces; *H* has as many rows *K* as signals are needed to control the external object, and as many columns *S* as there are body signals. Not being square, the matrix *H* does not have a unique inverse. But there exist infinite “right inverses” that combined with *H* yield the *K* x *K* identity matrix in the task space of external control signals. Each such right inverse transforms a desired position of the controlled object into one particular set of values for the body signals. We consider users to be competent when they are able to move their body successfully in response to a presented target for the controlled object. Mathematically, we consider this as finding one right inverse *G* of the mapping *H*, out of a multitude of possible and equally valid choices. Current theories and experimental observations [10] suggest that learning is a dynamical process in which the learners modify their behavior based on the errors observed at each iteration of a task. In the kinematically redundant conditions considered here, learning is problematic because a given low-dimensional task error signal has multiple representations in the high-dimensional body space. Here, we considered an error surface defined by the squared task error in the space of the elements of the target-to-body map *G* adopted by a learner, where *G* is the learner’s inverse model of the body-to-cursor mapping *H* established by the BoMI.

We implemented our learning model based on the hypothesis that the learners update the map *G* by moving along this error surface, following the line of steepest descent determined by the gradient of the squared error with respect to the elements of *G*. This error gradient depends on several variables; some can be directly observed by the learner, such as the error made in attempting to reach a given target position of the external device. However, the error gradient also depends upon the elements of the interface map *H*, which the learner cannot be assumed to know. Therefore, gradient descent learning of the inverse model requires a concurrent learning of the forward model. The latter requires a different error surface, since the forward map relates body configurations to the consequent position of the controlled object. Forward model learning does not require a target position for the controlled object, as the relevant error in this case is the difference between predicted and observed position of the controlled object. The squared prediction error defines an error surface in the space of the elements of the estimated forward map $\hat{H}$.

Learning is thus described through two first-order dynamical processes determined by two state equations. A forward learning process:

$${\hat{H}}^{\left(n+1\right)}={\hat{H}}^{\left(n\right)}+\varepsilon (p^{\left(n\right)}-{\hat{H}}^{\left(n\right)}q^{\left(n\right)})q^{T(n)}$$ (1)

and an inverse learning process

$$G^{\left(n+1\right)}=G^{\left(n\right)}-\eta \;{\hat{H}}^{(n)T}\;e^{\left(n\right)}\;u^{T(n)}$$ (2)

where *H*^(*n*)^ is a *K*x*S* matrix, *G*^(*n*)^ is an *S*x*K* matrix, *p*^(*n*)^ and *q*^(*n*)^ are column vectors of respectively *K*x1 and *S*x1 elements, *ε* and *η* are scalars. In the experiments reported here, *S* = 8 and *K* = 2. The reaching error *e*^(*n*)^ = (*p*^(*n*)^ − *u*^(*n*)^) that guides this process is the difference between actual and desired positions of the controlled object. For details on the derivation of these equations, see Methods. The forward and inverse models are effectively the states of the respective learning processes, whose *n*-th iteration results in state variables ${\hat{H}}^{\left(n\right)}$ and *G*^(*n*)^.

Eq (1) updates the subject’s estimate $\hat{H}$ of the forward model *H* that transforms *S*-dimensional body configurations *q* into *K*-dimensional positions *p* of the controlled object. The term in parentheses is the prediction error between actual and predicted positions of the controlled object. The concurrent process described by Eq (2) is the learning of the inverse model *G* that the subjects use to map the target object position *u* onto body signals *q*. Two possibly different learning rates, *ε* and *η*, provide inverse time constants for the respective learning processes.

Since we focus on the case in which forward and inverse learning are carried out concurrently, naïve users are immediately presented with the reaching task, and as they practice they observe both the reaching error and the prediction error. Eqs (1) and (2) are coupled through ${\hat{H}}^{\left(n\right)}$ and through a third equation that describes the body signals currently adopted to reach the target position:

$$q^{\left(n\right)}=G^{\left(n\right)}u^{\left(n\right)}.$$ (3)

This apparently innocuous interaction has potentially harmful effects on the convergence of the coupled learning dynamics, as the second term in the gradient contribution to Eq (1) includes a quadratic factor in *q*^(*n*)^ and thus in *G*^(*n*)^. This contribution may result in local minima, a problem avoided by adding noise to Eq (3), to obtain:

$$q^{\left(n\right)}=G^{\left(n\right)}u^{\left(n\right)}+\xi^{\left(n\right)}.$$ (4)

We validated our approach with six healthy subjects that learned to control the two-dimensional movement of a cursor on a monitor using eight signals from their upper body motions (shoulders and upper arms on both sides, see Methods for details). In these experiments, *S* = 8 and *K* = 2, and the state space of the combined forward-inverse learning was 2x16 = 32-dimensional.

### Dynamics of learning in human subjects

We monitored the learning process through two scalar metrics: RE, the L2 norm of the reaching error (the difference between actual *p*^(*n*)^ and target *u*^(*n*)^ location of the cursor at the end of each reaching movement), and IME, the spectral norm of the inverse model error (the difference between the identity matrix *I*_*K*_ and the product between the interface map *H* and the current estimate *G*^(*n*)^ of the inverse model). The spectral norm of a matrix, indicated here by ∥·∥ to emphasize its analogy with the L2 norm of a vector, is the maximum singular value of the matrix.

We estimated *G*^(*n*)^ from target and body signal data by least squares fit on Eq (3). The elements of *G*^(*n*)^ were estimated using data from 12 trials: trial *n* and its 11 preceding trials. Overlapping moving windows that included 12 trials were shifted by one trial at each iteration.

**Fig 1:**
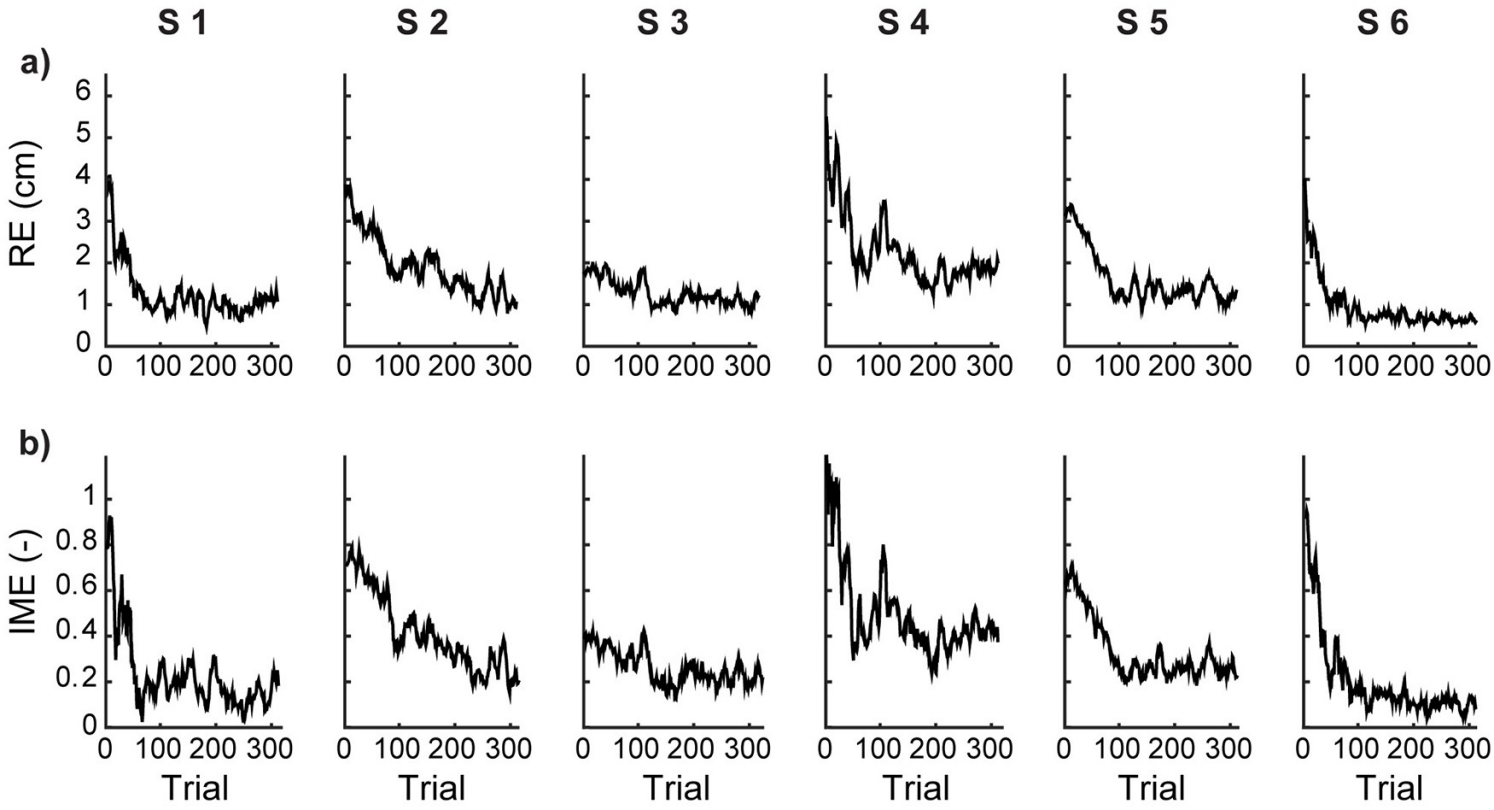
Subjects learn to use the body-machine interface. Data for the six subjects enrolled in the study (S1- S6). (**a**) Temporal evolution of the norm RE of the reaching error, calculated over a moving window that includes the current and the 11 preceding trials. (**b**) Temporal evolution of the norm IME of the inverse model error. The inverse model ***G***^(***n***)^ was obtained by a least squares fit on Eq (3) from target and body signal data for the current and the 11 preceding trials.

**Fig 2:**
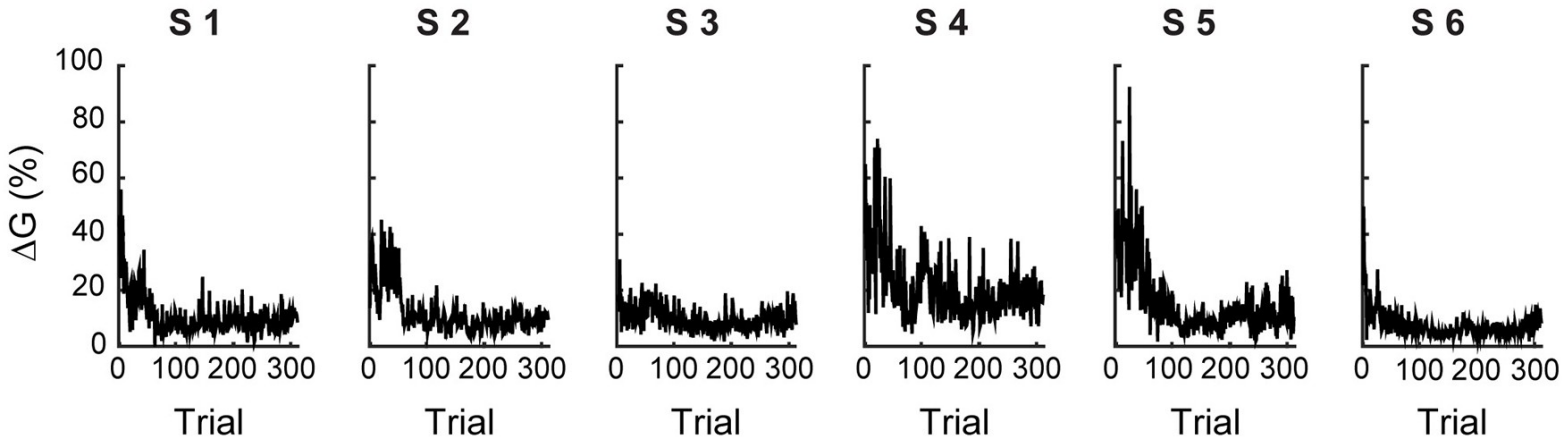
Temporal evolution of the changes in the inverse map *G*^(*n*)^. Changes in the inverse map as a function of trial number ***n***, quantified by ∥**Δ*G***^(***n***)^∥ = ∥***G***^(***n***)^ − ***G***^(***n*−1**)^∥⁄∥***G***^(***n***)^∥ (see Eq (19) in Methods), for the six subjects enrolled in the study (S1-S6).

In the experiment, each subject practiced with a personalized body-to-cursor map, chosen to reflect the statistics of its own freely produced upper body motions (see calibration procedure in Methods). With increasing number of practice trials, their RE decreased to values closer to the target radius (1 cm, Fig 1a). The learning process took over one hundred steps (172 ± 32 trials, mean ± SEM) before reaching asymptotic performance (Fig 1a), identified as the time when the norm of the reaching error was smaller than the radius of the target. Similarly, the matrix *G*^(*n*)^ converged to a generalized inverse of the body-to-cursor map (Fig 1b). Although the subjects explored a number of different body configurations while learning how to control the cursor, in the end they found a stable movement pattern, and built an inverse model *G*. Note the asymptotically small variations in the acquired *G*, with ∥Δ*G*∥ about 10% of ∥*G*∥ (Fig 2).

For each subject, both RE and IME errors decreased with time following a trend captured by an exponential curve (Eq (22)). The learning rates, each given by the inverse of the time constant of the corresponding exponential fit, are shown in Table 1 for each subject. Note the great similarity of these two rates for any given subject.

**Table 1:** Exponential rate used to best approximate the decay of RE (*λ*_*RE*_) and IME (*λ*_*IME*_) with *n*, respectively. The R^2^ values quantify the goodness-of-fit of the exponential model for each subject to the corresponding experimental data.

Subject	RE		IME	
	*λ*_*RE*_	$R_{RE}^{2}$	*λ*_*IME*_	$R_{IME}^{2}$
S 1	0.036±0.003	0.87	0.036±0.004	0.78
S 2	0.010±0.001	0.85	0.006±0.001	0.86
S 3	0.015±0.003	0.73	0.014±0.003	0.75
S 4	0.016±0.003	0.80	0.022±0.004	0.80
S 5	0.021±0.001	0.87	0.020±0.002	0.86
S 6	0.029±0.002	0.93	0.029±0.002	0.90

### Learning dynamics: Model vs. human subjects

To build a model for each subject, we used the subject-specific map *H* and the subject-specific sequence of targets used for training. The learning rate *η* for the inverse model (Eq (2)) was taken to be equal to the subject-specific rates *λ*_*RE*_ reported in Table 1 (see Methods), obtained for each subject by fitting with standard least squares an exponential decay curve to the corresponding experimental time series of reaching errors (Fig 1a) to extract the decay rate *λ*_*RE*_. The learning rate *ε* for the estimation of the forward model (Eq (1)), and the amplitude *σ* of the noise added to the inference of body motions (Eq (3)) followed from an optimization procedure (see Methods). Table 2 reports the values of these three parameters for each subject. We let the model evolve until the norm of the reaching error was smaller than 1 cm, as the subject’s performance reached a plateau once the cursor reached the target.

**Table 2:** Subject-specific model parameters. The learning rates *η* and *ε* correspond to the inverse and the forward model, respectively; *σ* is the amplitude of the Gaussian noise added to the inference of target-specific body configurations.

Subject	*σ*	*ε*	*η*
S 1	0.7335	0.1774	0.036
S 2	0.6587	0.2463	0.010
S 3	0.7395	0.1924	0.015
S 4	0.7241	0.2178	0.016
S 5	0.8278	0.1540	0.021
S 6	0.7794	0.1937	0.029

**Fig 3:**
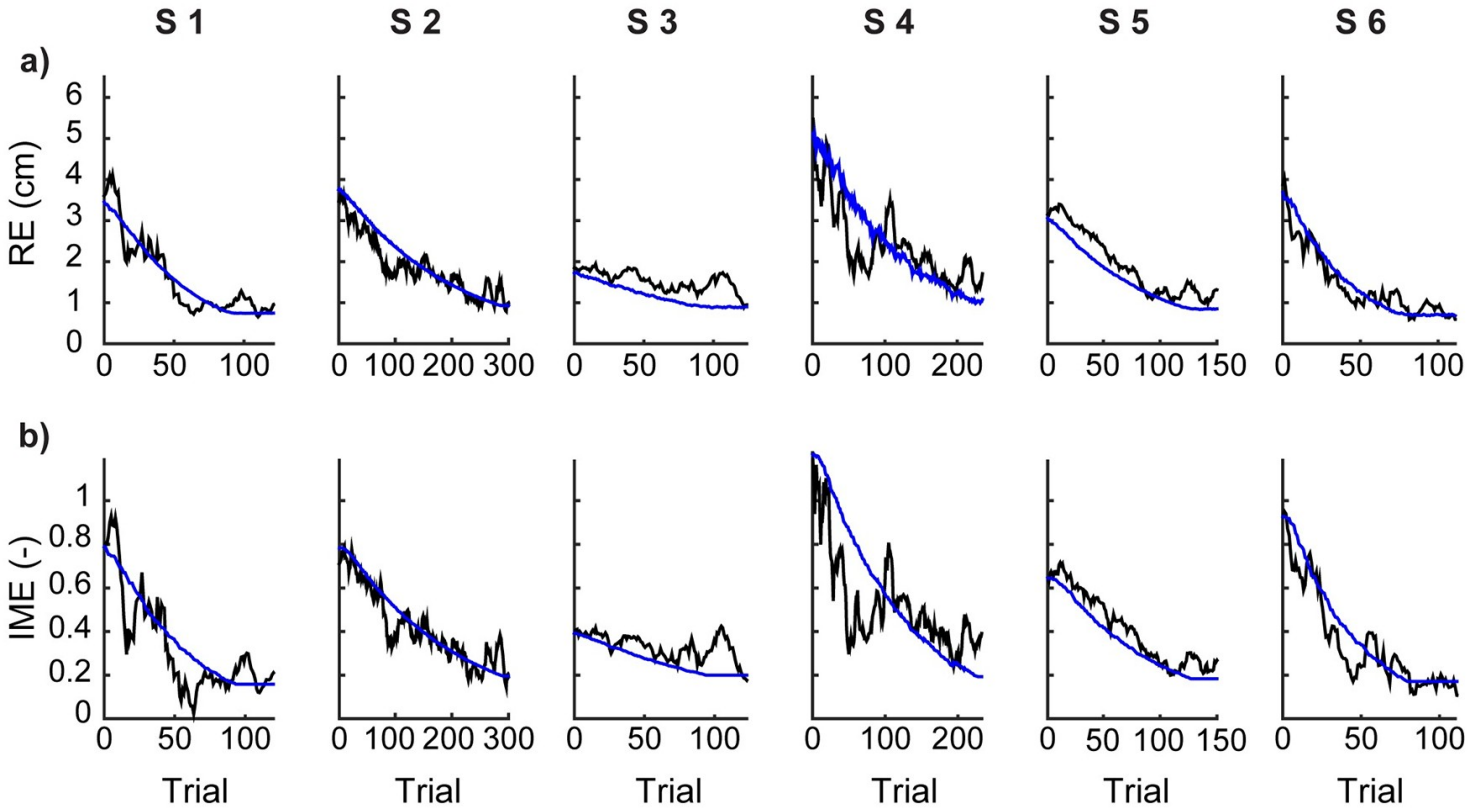
Modeling human learning. (a) Temporal evolution of the norm RE of the reaching error as a function of trial number ***n***, calculated from the data of six subjects (black) and from the respective models (blue). (b) Temporal evolution of the norm IME of the inverse model error as a function of trial number *n*, calculated from the experimental data (black) and from the model simulation (blue). Both metrics are calculated over a moving window that encompasses the current and its 11 preceding trials.

We then tested how well our model captured the learning dynamics of each subject. As shown in Fig 3, these subject specific models were able to predict quite well the individual experimental learning curves. Both the RE and the IME estimated from the model follow the time evolution extracted from the real experimental data. Correlation coefficients (Table 3) quantify the similarity between the simulated and actual temporal evolution of RE and IME during learning.

**Table 3:** Correlation coefficients (R^2^) between the temporal evolution recorded during the experiment and the temporal evolution predicted by the model, for both the norm RE of the reaching error and the norm IME of the inverse model error, for each subject (S1-S6).

Subject	Correlation RE	Correlation IME
S 1	0.93	0.87
S 2	0.90	0.90
S 3	0.78	0.78
S 4	0.77	0.70
S 5	0.98	0.97
S 6	0.89	0.83

We investigated the stability of the simulated RE and IME curves against changes in the noise parameter *σ*. Simulations for each subject using the learning rates *η* and *ε* listed in Table 2 revealed that increasing *σ* by up to 0.1 and decreasing it by up to 0.2 around the optimal values listed in Table 2 did not affect the good fit of the simulated curves to the experimental ones. Further decreases in *σ* affect model performance because the amplitude of the noise becomes insufficient to free the gradient descent algorithm from getting trapped in local minima. Further increases in *σ* affect model performance because the exploration of the state space becomes dominated by noise instead of being guided by gradient descent.

In principle, learning of the forward map and its inverse could take place in two separate phases. First, a phase guided by a comparison between actual object position and expected object position; their difference results in prediction errors that drive the estimation of the forward model. Second, a phase guided by a comparison between actual object position and target; their difference results in reaching errors that drive the acquisition of the inverse model. This two-phase mechanism leads to the model learning curves shown in S1 Fig (blue curves). The two-phase model does not fit the experimental data as well as a model of forward and inverse learning as concurrent processes, shown in Fig 3 (blue curves). These results support our approach of modeling forward and inverse learning as concurrent processes.

**Fig 4:**
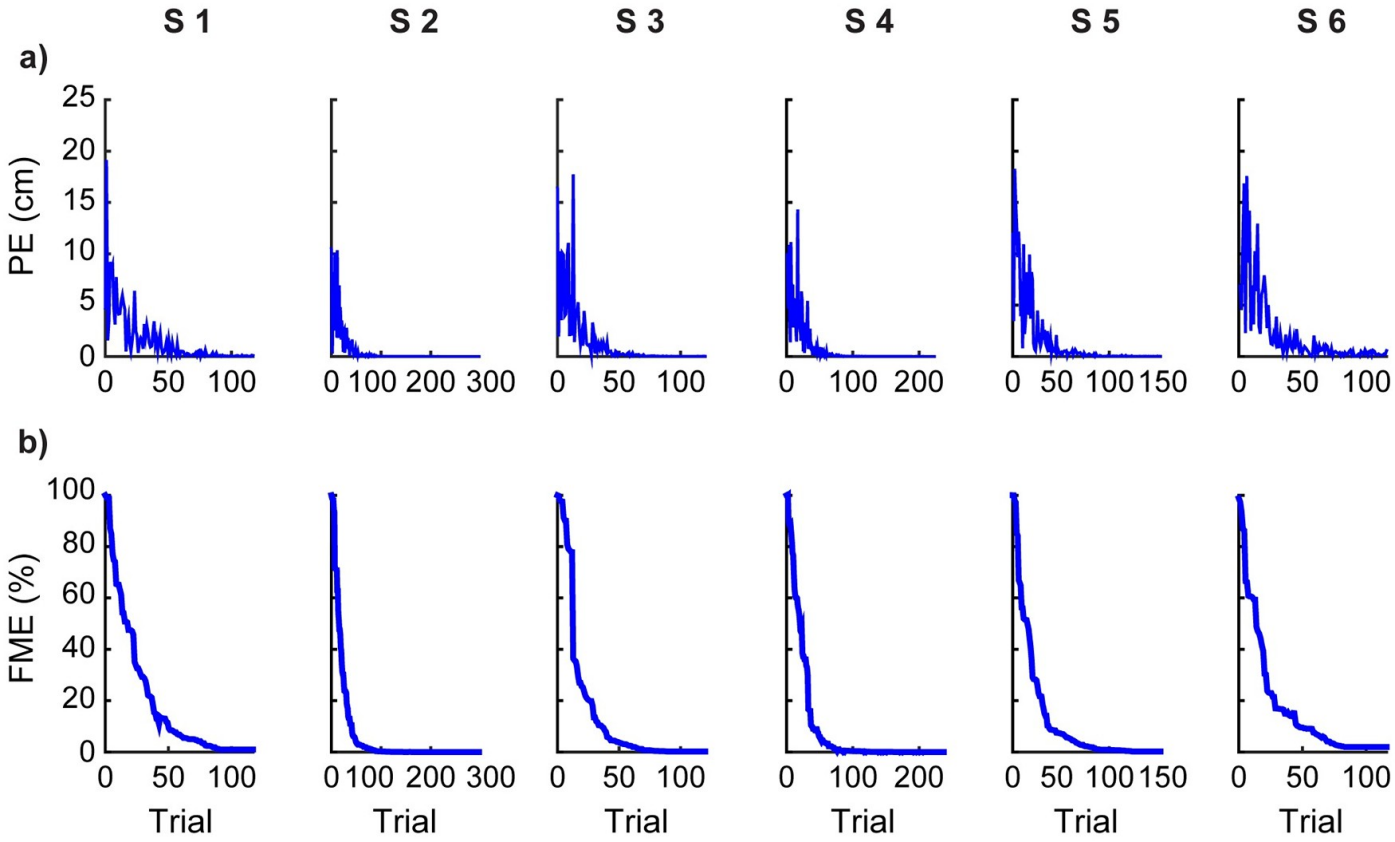
Subjects learn the forward model: Data from model based simulations of the temporal evolution of the forward map estimate $\hat{H}$. **(a)** Temporal evolution of the norm PE of the prediction error as a function of trial number *n*. **(b)** Temporal evolution of the estimate of the forward map $\hat{H}$, quantified by $FME=∥{\hat{H}}^{\left(n\right)}-H∥/∥H∥$, as a function of trial number *n*. Data are shown for each subject-specific model. Only the first 100 iterations of the dynamics are presented, since the asymptotic regime has by then been reached for both metrics.

The model also allows us to compute a current estimate of the forward map $\hat{H}$ as acquired by the subjects while practicing. We quantified the similarity between the estimated ${\hat{H}}^{\left(n\right)}$ and the BoMI map *H* at each iteration of the learning dynamics by using the forward model error (FME), defined as the spectral norm of the difference between *H* and ${\hat{H}}^{\left(n\right)}$, normalized by the spectral norm of *H* (see Eq (20) in Methods). This error in the estimate of the map that transforms body configurations into cursor position leads to the cursor prediction error, the difference between the actual and the expected position of the cursor. We monitored the norm PE of the prediction error and the FME as a function of trial number *n* (Fig 4). The estimate ${\hat{H}}^{\left(n\right)}$ converged toward the actual forward map *H*, resulting in near zero asymptotic values for both PE and FME (Fig 4).

**Fig 5:**
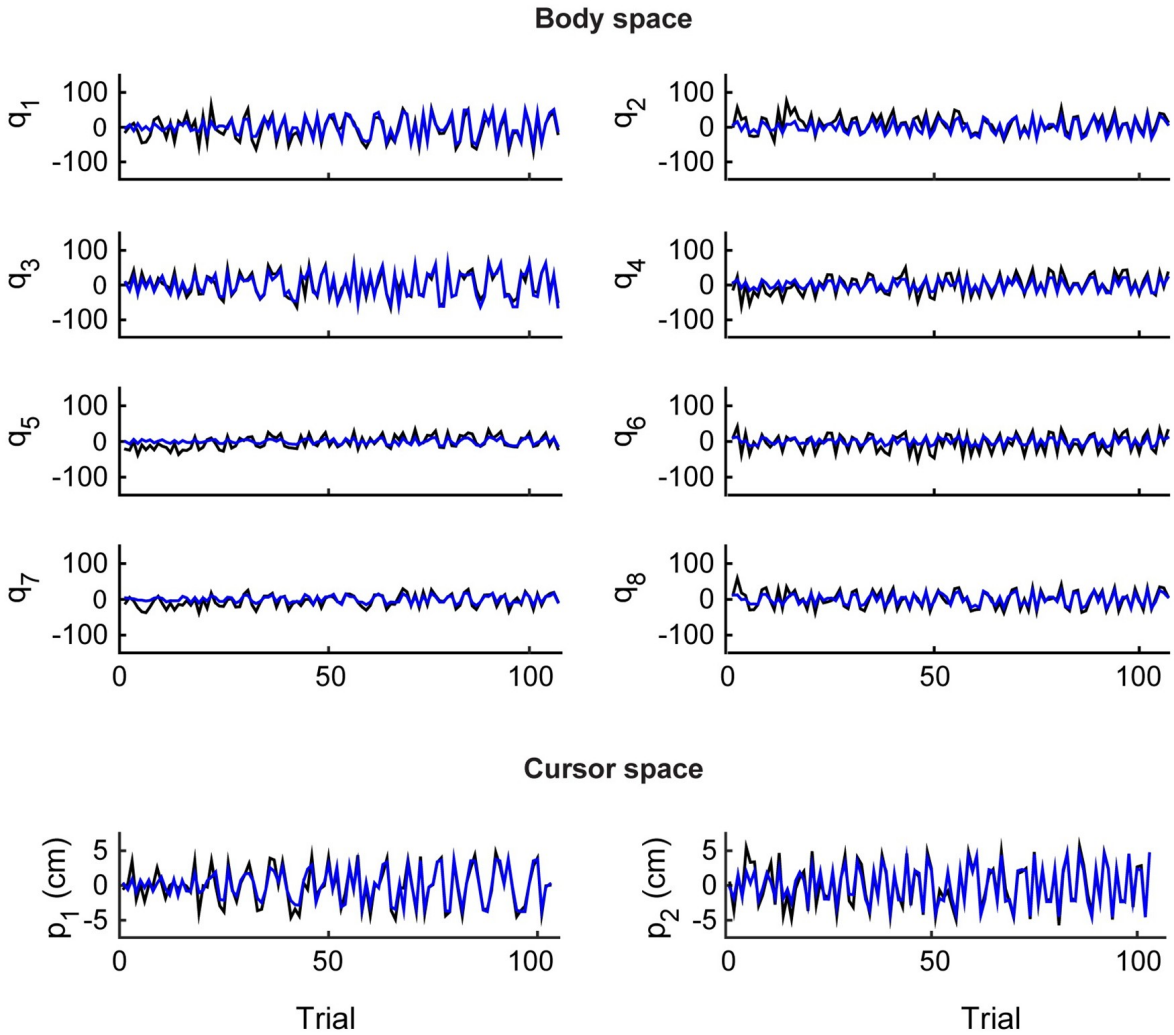
Reconstruction of body and cursor signals. Comparison between real (black) and simulated (blue) data for subject 6. The top panel presents the values of the eight body signals (q_1,_ …, q_8_), i.e., the (x,y) coordinates of four markers (shoulders and upper arms on both sides) in the image plane of the associated cameras; the bottom panel shows the (x, y) coordinates of the cursor (p_1_, p_2_) in the reference frame of the computer monitor.

The dynamics of the learning model captured the errors in the low-dimensional task space of the controlled cursor as well as the history of body signals generated by the subjects in response to the successive targets (Fig 5 and Table 4); the sole exception was Subject 4, whose accuracy in reaching the target position was smaller and characterized by a higher level of variability (Fig 3a). The body and cursor signals recorded during the experiment and predicted by the model were not very similar at the beginning of training, but they quickly converged and tended to overlap by the time RE and IME reached their asymptotic condition (Fig 5, Table 4). These results support the conclusion that a model of learning based on simple gradient descent over the two quadratic error surfaces defined here captures the formation of forward and inverse representations of the map established by the body-machine interface.

**Table 4:** Correlation coefficients (R^2^) between the temporal evolution of the actual signals recorded during the experiment and the temporal evolution of these quantities as predicted by the model, for both body signals (q_1_, …, q_8_) and cursor coordinates (p_1_, p_2_). Only the last 100 trials were used to compute the R^2^ values.

Subject	Body space	Cursor space
S 1	0.90	0.96
S 2	0.90	0.97
S 3	0.93	0.98
S 4	0.69	0.93
S 5	0.91	0.98
S 6	0.93	0.99

## Discussion

We investigated the learning process that occurs when subjects reorganize or "remap" their body motions as they learn to perform a task that involves a novel relation between body motions and their observable consequences. For a patient who suffers from severe paralysis and is obliged to reorganize the available mobility to operate an assistive device such as a powered wheelchair through a human-machine interface, the ability to engage in such remapping becomes a necessity of life. Here we investigated the process of learning to perform reaching movements via a body-machine interface; we used a group of unimpaired subjects under the preliminary assumption that similar learning mechanisms are present in people suffering from injuries to the spinal cord. We considered a body-machine interface that harnesses signals generated by body configurations in order to control an external object, in this case the position of a cursor on a computer monitor. The interface establishes a many-to-one body-to-object map that the user must learn to master starting from a naïve state. The map is in fact not intuitive, as there is no obvious correspondence between the motions of the body and the motion of the controlled object.

The goal of our experiment was to test the hypothesis that motor learning while using a BoMI is a state based process with first-order deterministic dynamics, and that such computational model is suitable to describe both the estimation of the forward body-to-object map and the construction of its inverse by the unimpaired subjects involved in the experiment. Subjects trained to control a computer cursor through a BoMI whose linear body-to-object map was customized to each subject through a calibration procedure based on the statistics of the subject’s own body motions. Through practice with a fixed BoMI, all subjects demonstrated exponential convergence toward an inverse of the BoMI mapping, with subject-specific learning rates. The parameters of this inverse model define a state space in which learning is modeled as a first-order dynamical process that evolves based on the specific sequence of target positions for the external object, and of two types of error: (i) the prediction error that is the difference between the actual position of the object and the position predicted by the subjects based on their internal representation of the interface map, and (ii) the task error or reaching error that is the difference between the position reached by the object and the actual target position. While the prediction error depends on the current estimate of the forward model, the reaching error depends on the current state of the inverse model, which determines the chosen body configuration.

We studied the evolution of the learning system using different interface maps *H*, learning rates *η*, and target sequences *U* for each subject. The empirical observations of human adaptation to the BoMI were compared to predictions from a subject-specific learning model that used initial conditions inferred from each subject’s initial performance, the same *H* and *U* as used by the subject, and a learning rate *η* obtained by temporal regression of the experimental data. We demonstrated that a model based on first-order dynamics was sufficient to capture the evolution of learning as described through both the observed errors and the accuracy of the estimated internal models. There was however a notable difference between the models’ and the subjects’ learning: the actual decay of the norm of the reaching error computed from experimental data was not as smooth as the decay predicted by the model. This might be due to the main simplifications adopted in our model, namely, i) the learning dynamics (Eqs (1) and (2)) were assumed to be deterministic, and ii) the learning of the inverse model (Eq (2)) was assumed to be linear.

The comparison between model predictions and actual data in Fig 3 indicates that our proposed model of learning is sufficient to explain the data. However, the mechanism we propose is not necessary; we cannot rule out other possibilities, such as reinforcement learning. The approach adopted here, in which learning occurs via a gradient descent search to decrease the reaching error over the space of parameters of the inverse model *G*, requires knowledge of the forward map *H* to compute the gradient (see Methods, Eqs (9), (10) and (11)). We considered it not plausible that naïve learners would possess such knowledge. Thus, we hypothesized that subjects learn an estimate $\hat{H}$ of the forward map *H* concurrently with their acquisition of an inverse map *G*. In earlier work Sanger (2004) [24], proposed a computational model for estimating the user’s inverse model *G* that avoided the need for information about the forward map *H* through a direct approximation of the user’s response *q* to a presented target *u*. Our work develops these ideas further, with the goal of estimating not just the user’s inverse model but the learning process that leads to the formation of the inverse model. To estimate the inverse model, Sanger proposes an algorithm based on an error *δ* = q* − *G*(*u*), where q* is one of the many responses that satisfy *H*(q*) = *u* and would allow the user to achieve the target. Note that q* is not observable by the user. In contrast, the learning process that we model is based on quantities that the user can observe: body actions, the outcome of those actions, and their targets. In our model of the user’s learning process, the user must have information about the forward map to learn its inverse.

Our calculations of the two metrics that quantify the learning of $\hat{H}$, the prediction error PE and the forward model error FME, are shown in Fig 4. These metrics have been obtained from the model and are not being compared to results based on experimental data. However, the estimator $\hat{H}$ gets folded into the learning of *G*. The agreement between the results from the model and those obtained from experimental data are shown in Fig 3 for the two metrics that quantify the learning of *G*, the reaching error RE and the inverse model error IME. Since the model is based on the concurrent learning of *G* and $\hat{H}$, we argue that this agreement is not only an explicit validation of the acquisition of a valid inverse model, but also an implicit validation of the concurrent and successful estimation of the forward model. This agreement between model and experimental results does not exclude the possibility of alternative learning mechanisms, such as a direct learning of the inverse model [24] or the use of reinforcement learning [25] to acquire an action policy that would play the role of the inverse model.

Although the interface forward map is linear (Methods, Eq (5)), this is a many-to-one map admitting a multitude of inverses. This “redundancy” opens the possibility of successful linear and nonlinear inverse maps. Redundancy also leads to an important consideration about gradient descent learning. The reaching error surface in the space of the inverse model elements does not have a unique minimum, but a continuously connected set of minima corresponding to the null space of the forward map. In the metaphor of a skier descending from a mountain following the gradient, this space of equivalent inverse models corresponds to a flat elongated valley at the bottom of the mountain. Anywhere along the valley is a valid end to the ride, as it corresponds to a valid inverse model. The inverse model on which the steepest descent ends depends on the initial conditions, as predicted by the dynamical model (see Fig 3b–evolution of the norm of the inverse model error), as well as on the realization of the noise employed in any given simulation of the learning model. On the other hand, there is only one valid solution for the estimate of the forward model. Our simulations of the model reach the correct solution for $\hat{H}$, as demonstrated by the convergence to zero of the prediction error and of the norm of the forward model error in Fig 4.

The analysis of the learning dynamics of the users of a body-machine interface is essential for the effective development of coadaptive approaches, where the interface parameters are themselves updated based on the user’s state of learning [26–28]. Coadaptation requires a seamless integration between machine learning and human learning. Both learning processes are dynamic, evolving as functions of their own internal state and of inputs that reflect the state of their counterpart. A mismatch between the timing of the interface updates relative to the subject’s learning dynamics would likely lead to hindering human learning (if the interface update rate is too fast) or to ineffectiveness (if the interface update rate is too slow).

In our current understanding, motor learning is not only a way to acquire or improve a skill, but is perhaps most importantly a biological mechanism to gain knowledge about the physical properties of the environment [9]. Through the practice of movements, the brain learns to separate unpredictable from predictable features of the world in which the body is immersed; through the formation of representations or "internal models" of the predictable features, the brain acquires the ability to anticipate the sensory consequences of its commands. The human operator of a BoMI must develop an inverse model of the forward map to transform a desired goal into an action of the body, as demonstrated by previous studies where subjects operating the BoMI were able to compensate for the effects of visuomotor rotations, changes of scale, and noise perturbations applied to the cursor in the task space [29, 30]. Further evidence from the literature indicates that adaptive, error-driven, internal model formation is a general feature of motor learning, observed during arm reaching and drawing, and during pointing with the legs and walking [31].

Theoretical studies of motor learning have focused on control policies and internal models to understand how the brain generates action commands [32]. Control policies allow the brain to select goals and plan actions, while internal models generate motor commands that are appropriate for those plans in the context established by sensory feedback. For example, when the goal is to reach a target, the brain must first evaluate the current position of the limb with respect to the target and use a control policy to plan a movement of the hand [3, 33]. An internal model of the limb dynamics, called an inverse model, then converts that plan into motor commands [34–36], while a forward model of the same limb dynamics predicts the sensory consequences of these motor commands [14, 37, 38]. The comparison of this prediction with actual sensory feedback [39] allows for a re-estimation of the current hand position with respect to the target, and for an update of the motor plan [40] by issuing an error-dependent motor command aimed at correcting the ongoing movement. Forward and inverse internal models thus play a fundamental role in movement planning and execution.

Recent studies have considered the formation of these internal models as dynamical processes [10, 41, 42]. For example, to account for findings observed when reaching arm movements are perturbed by external force fields, Donchin and coworkers [10] argued that the forces generated by the subjects to compensate for the external field are the output of an internal model of the field, developed through experience. Their theory, successful at predicting the time history of adaptation, was based on two key assumptions that we also adopted here, namely i) that the movement outcomes and the ensuing errors result from a deterministic process, and ii) that the parameters of the internal model define the state space of the learning dynamics.

The transformation from the movements of the BoMI user to the movements of the controlled objects establishes a new geometrical relation between body motions and their consequences. The BoMI thus essentially creates a novel geometry that the user must learn to operate. Here, we have implemented a linear transformation from body signals to a cursor; this allowed us to work under the assumption that the users would develop a linear inverse model of this map. However, linearity of the inverse map is not a necessary consequence of operating through a linear forward map, because a linear forward map that is not bijective may also admit nonlinear inverses. Therefore, our approach will not result in the most general solution to the problem of finding an inverse map. Nevertheless, our analysis demonstrated that the linear inverse model derived by coupled gradient descent on both the prediction error and the reaching error is capable of reproducing with high fidelity the entire history of a subject’s responses to a sequence of targets (Fig 5). While we are unable to exclude more complex processes that could lead to an equally effective nonlinear inverse model of the linear BoMI map, the linearity assumption not only leads to results that agree with the experimental data but also fulfills Occam’s razor criterion for simplicity.

Issues of cognitive strategies that are involved in motor learning are important topics, in particular the decision processes that establish the balance of exploration and exploitation. However, our contribution to these topics can only be speculative. In our model there are two elements that drive the dynamical process: the errors {*e*^(*n*)^} and the inputs {*u*^(*n*)^}; in the reaching task, these correspond to reaching errors and to the targets presented to the subject. One might take the view of Taylor et al. [43] that “the contribution of explicit learning would be modulated by instruction and the contribution of implicit learning would be modulated by the form of error feedback”, and be tempted to conclude that these two terms are represented in our model by the inputs and the errors, respectively. However, our model prevents such a clear separation, since these two variables are closely correlated; even when the choice of body action in response to an input is distorted by noise, the error is directly connected to the input by the forward map *H* and the inverse model *G*^(*n*)^. Our model provides a mechanistic view of learning as a process in which the formation of the internal model is driven by the inputs through exposure to a target sequence. The separation of the effects associated with implicit and explicit learning might only be investigated by perturbing the connection between error and input established by the evolving inverse model.

We conclude with some comments on the clinical relevance of this study. Damage to the spinal cord, stroke, and other neurological disorders often cause long-lasting and devastating loss of motion and coordination, as well as weakness and altered reflexes. In most cases, some residual motor and sensory capacities remain available to the disabled survivor, and can be harnessed to provide control signals to assistive devices such as robotic systems, computers, and wheelchairs. A first challenge for the disabled [44] is to learn how to interact with the assistive devices and how these respond to the user’s actions.

A broad spectrum of sensors, such as inertial measurement units (IMUs) placed on the head [45] or electroencephalography (EEG) systems [46], are available for detecting and decoding movement intentions. A body machine interface (BoMI) captures residual body motions by optical [18–20], accelerometric [15, 17], or electromyographic sensors [47], and maps the sensor signals onto commands for external devices such as powered wheelchairs [17] or drones [20], or onto computer inputs. At the other end of the spectrum, brain-machine interfaces decode motor intention from neural activity recorded in motor or premotor cortical areas [22, 23, 48]. Both brain- and body-machine interfaces take advantage of the vast number of neural signals and degrees of freedom of the human body [1, 49, 50], and of the natural ability of the motor system to reorganize the control of movement [4, 9, 51, 52]. These interfaces establish a map—most often linear—from the space of neural or motion signals to the lower dimensional space of control signals for the external device [15, 53]. The user’s ability to operate the interface is expected to change over time; either a positive change associated with the acquisition of greater control skills, or a negative change due to the worsening of the user’s medical condition. In either case, the interface map also needs to change, to coadapt with its user. This coadaptation is a critical challenge in the development of both brain- and body-machine interfaces [26, 28]; harmonizing the interface update with the processes that guide the improvement or decay of the user’s skill is of obvious importance. Understanding the dynamics of human learning through the interaction with the interface carries the promise of creating truly intelligent systems, capable of compensating for the changing abilities of their users [22, 27].

## Methods

### Ethics statement

All experimental procedures were approved by the Northwestern University Institutional Review Board. All subjects signed a consent form prior participating to the study.

### Computational model

Inverse kinematics is a well-known and well-explored computational problem in robotics [54, 55] and human motor control [34, 56]; it refers to finding the configuration of joint angles that results in a desired position of an end effector or of the hand in the operational space [57]. Inverse kinematics problems become ill-posed [58] when there are multiple valid solutions as a consequence of the many-to-one nature of the forward kinematic map. This is the situation considered here, in which the kinematics that the subjects are controlling may be partitioned in a sequence of two maps. In a first map, the subjects control the motions of their bodies by acting on a multitude of muscles and joints. In a second map, the signals triggered by these body motions determine the lower dimensional motion of an external object such as a wheelchair [17], a cursor on a computer screen [16], or a drone [20]. Here we make the critical but reasonable assumption that the subjects have already acquired in a stable form the expertise needed to control the motion of their body, or at least portions of it that were unaffected by injury or disease. Therefore, they only need to acquire the second component. This is the component we focus on, limited here to a body-machine interface whose linear map *H* transforms, at any given trial *n*, an *S*-dimensional vector of body signals *q* into a *K*-dimensional control vector *p* as follows:

$$p^{\left(n\right)}=Hq^{\left(n\right)}=[\begin{array}{ccc} h_{1,1} & \cdots & h_{1,S}\\ \vdots & \ddots & \vdots \\ h_{K,1} & \cdots & h_{K,S} \end{array}]q^{\left(n\right)}$$ (5)

Here, *H* is a *K*x*S* matrix. Since *K*<*S*, this interface map is many-to-one and there is a "null space" of inputs for each value of the output, encompassing all different patterns of body signals that result in the same control signal. This is an important characteristic of the map; earlier work has shown that subjects learn through practice to separate the null space from its orthogonal “potent space” complement [53].

#### Learning dynamics as first-order state-based model

In a learning experiment where the goal is to reach targets in the control space, the superscript *n* labels the trials or successive repetitions of a single action; for instance, each trial is a reaching movement in a sequence of such movements. At the end of a trial, the learner observes an error *e*^(*n*)^. This error drives the updating of the internal inverse model, which we assume to be a linear map *G*^(*n*)^ transforming a goal *u*^(*n*)^ into its corresponding body vector (previously Eq (3)):

$$q^{\left(n\right)}=G^{\left(n\right)}u^{\left(n\right)}$$ (6)

Since the forward map *H* is linear, the linearity of *G* is a sufficient but not necessary condition. More complex, nonlinear forms of the inverse model would in principle be admissible. Here, we assume the simplest general form for a linear inverse model of the forward BoMI map; this assumption makes the investigation of learning dynamics tractable.

For the *n*-th reaching trial, *u*^(*n*)^ is the position of the target and the reaching error is the *K*-dimensional vector from the target position to the actual position of the controlled object at the end of the trial:

$$e^{\left(n\right)}=p^{\left(n\right)}-u^{\left(n\right)}.$$ (7)

The internal model thus becomes a right-inverse model of the interface map:

$$e^{\left(n\right)}={p^{\left(n\right)}-u^{\left(n\right)}=(HG}^{\left(n\right)}-I_{K})\;u^{\left(n\right)}.$$ (8)

As learning reaches a steady state, participants are expected to have eliminated this error. At this point, *e*^(*n*)^ = 0, which requires *H G*^(*n*)^ = *I*_*K*_. Note that the reaching error, a *K*x1 vector, can be interpreted as the projection of the *K*x*K* matrix (*HG*^(*n*)^ − *I*_*K*_) onto the *K*x1 vector *u*^(*n*)^. The metrics introduced earlier to monitor the acquisition of the inverse model are RE, the L2 norm of the *K*x1 reaching error (*HG*^(*n*)^ − *I*_*K*_) *u*^(*n*)^, and IME, the spectral norm of the *K*x*K* matrix (*HG*^(*n*)^ − *I*_*K*_).

Learning is represented as a dynamical process whose state is the internal inverse model *G*^(*n*)^; this state changes after the observation of each reaching error. The targets presented to the learner constitute the external input to this process. To ensure that the change in state leads to a reduction of the error, the learning process drives the state along the gradient of the quadratic error surface in the state space defined by the components of *G*. The gradient of the squared reaching error with respect to the components of the inverse model is

$$\nabla_{G}\;\frac{1}{2}{∥(H\;G^{\left(n\right)}-I_{K})\;u^{\left(n\right)}\;∥}^{2}=H^{T}(H\;G^{\left(n\right)}-I_{K})\;u^{\left(n\right)}u^{T(n)},$$ (9)

which leads to the update equation

$$G^{\left(n+1\right)}=G^{\left(n\right)}-\eta \;H^{T}(H\;G^{\left(n\right)}-I_{K})\;u^{\left(n\right)}\;u^{T(n)},$$ (10)

or, equivalently

$$G^{\left(n+1\right)}=G^{\left(n\right)}-\eta H^{T}e^{\left(n\right)}u^{T(n)}.$$ (11)

Here, *η* is a learning rate parameter that we model as a scalar. Although in principle there could be a different rate for learning every element of the forward and inverse models, we found that only two learning rates, *ε* for the forward model and *η* for the inverse model, sufficed to account for the observed learning behavior.

If the interface map *H* is known, the update Eq (11) provides an estimate of the right inverse of *H* solely on the basis of control space data, without performing an explicit matrix inversion. Given the targets *u*^(*n*)^, the variables of interest are the observed reaching errors *e*^(*n*)^ and the estimated inverse model or "state of learning" *G*^(*n*)^. As *e*^(*n*)^ → 0, *G*^(*n*)^ becomes stationary.

The gradient of the error involves the actual value of the interface map *H*. It is not plausible to assume that our subjects had any initial notion of the interface map, let alone an exact representation. In a realistic model of learning, the value of *H* must be replaced with an evolving estimate ${\hat{H}}^{\left(n\right)}$. In this scenario, the current state of learning is represented in a higher dimensional space that includes the components of both *G*^(*n*)^ and ${\hat{H}}^{\left(n\right)}$. In the case of *S* = 8 body signals controlling the location of an object in *K* = 2 dimensions, the state space for learning is 2x8x2 = 32-dimensional. We follow a concept introduced by Jordan and Rumelhart [14], and represent learning as the parallel development a of forward-inverse model. The forward model leads to a prediction of the controlled device position given the current set of body signals, whereas the inverse model generates the body signals needed to achieve a given target position of the device.

In our study, we distinguish between the exploration that takes place when subjects perform tentative movements as they are seeking a goal, and the performance of aimless limb motions, which we refer to as “motor babbling” in accordance with the literature on human and robotic development [59, 60]. In our experiments, babbling takes place during the dance calibration used to establish the forward map. Motor babbling has also been considered as an exploratory behavior leading to the formation of internal dynamical models. However, here we use the concept of “exploration” to refer to the search for alternative solutions to the problem of generating a body configuration *q* that makes it possible to reach the target *u*.

In principle, motor babbling and goal directed learning could take place in two separate phases. First, a motor babbling phase where freely produced, aimless body motions result in actual object motions that are compared to expected object motions to obtain prediction errors that drive the estimation of the forward model. Second, a phase where the subjects reach for specific targets, and the resulting reaching errors drive the estimation of the inverse model (see S1 Fig). This two-phase mechanism has been suggested as a possible model of motor development in infants [61], who acquire a model of the dynamical properties of limbs by driving them with erratic neuromuscular patterns of activity. However, this is not the case in experiments where subjects are presented with reaching targets from the onset; in this scenario, no aimless motor babbling was observed. It is thus more plausible to model forward and inverse learning as concurrent processes.

The prediction error quantifies the difference between actual and predicted positions of the controlled object, without reference to a target. The gradient of the squared prediction error with respect to the components of the forward model *H* is

$$\nabla_{\hat{H}}\;\frac{1}{2}{∥p^{\left(n\right)}-{{\hat{H}}^{\left(n\right)}\;q}^{\left(n\right)}∥}^{2}=-\;(p^{\left(n\right)}-{\hat{H}}^{\left(n\right)}\;q^{\left(n\right)})q^{T(n)}.$$ (12)

The update equations for the coupled learning process then are

$${\hat{H}}^{\left(n+1\right)}={\hat{H}}^{\left(n\right)}\;+\varepsilon \;(p^{\left(n\right)}-{\hat{H}}^{\left(n\right)}\;q^{\left(n\right)})q^{T(n)},$$ (13)

$$G^{\left(n+1\right)}=G^{\left(n\right)}-\eta {\hat{H}}^{(n)T}\;e^{\left(n\right)}u^{T(n)},$$ (14)

reported earlier as Eqs (1) and (2). Note that Eq (13) contains a term that is quadratic in *q*; this implies a quadratic dependence on the elements of *G*^(*n*)^, since at each step *q*^(*n*)^ is derived by applying *G*^(*n*)^ to the corresponding target. The update equation for the estimate of the forward model is thus not linear, and the learning process is prone to getting stuck in local minima. The simplest way to avoid this is to add a small amount of noise to the body signals derived from the inverse model,

$$q^{\left(n\right)}=G^{\left(n\right)}u^{\left(n\right)}+\xi^{\left(n\right)},$$ (15)

where the noise *ξ* has the same dimension *S* as *q*, and each component of *ξ* is independently drawn from a Gaussian distribution $N(0,\sigma^{2})$ at each trial.

Two important parameters of the combined learning model of Eqs (13) and (14) are the learning rates for the forward (ε) and the inverse (*η*) models. In the case of eight body signals mapped into a two-dimensional control space, these learning rates apply to the evolution of 16 elements each; their most general form would be a 2x8 matrix for *ε* and an 8x2 matrix for *η*. Here, for simplicity and to avoid overfitting, we assume both learning rates to be scalar. A similar assumption is made for the noise amplitude *σ*, a somewhat less critical parameter whose sole purpose is to add sufficient noise to the learning algorithm so as to avoid getting trapped in local minima.

### Validation of the model with experimental data

To validate the outcomes of the model we recruited six unimpaired subjects (age range 21–40 years old, 3 males and 3 females) in the preliminary study.

**Fig 6:**
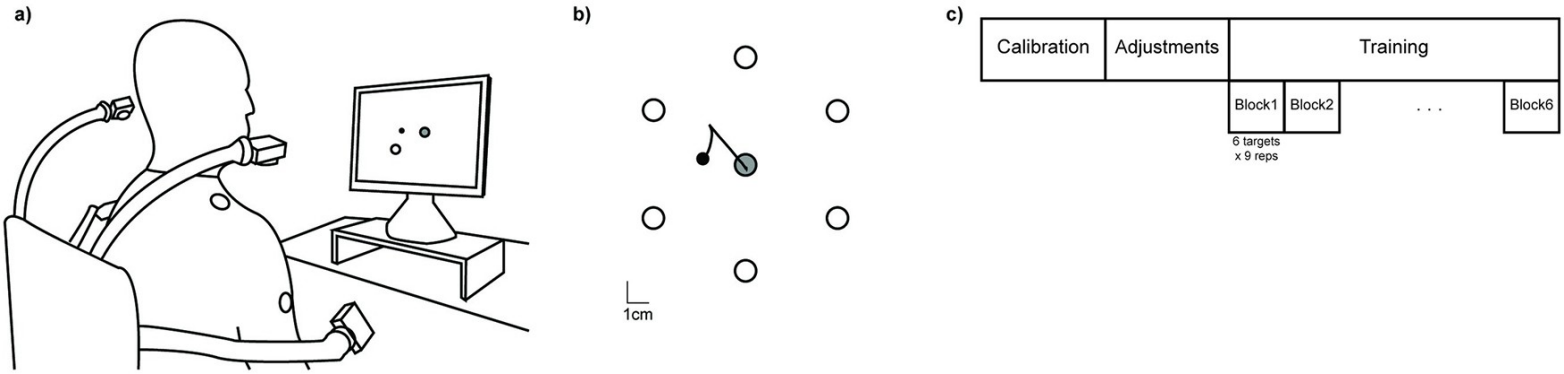
Experimental setup and protocol. **a)** Schematic overview of the setup of the Body Machine Interface. The subject sat on a chair in front of the computer. Active optical markers were placed on shoulders and upper arms; their positions were recorded by infrared cameras. **b)** Design of the reaching task. Six targets were equally spaced over a circumference of 5 cm radius, centered in the computer screen. The grey circle corresponds to the central target; this was the initial position from which each movement towards the peripheral targets (empty circles) would start; movements would return to the central target after the peripheral target was reached. The black small circle is the cursor controlled by the subjects. **c)** Experimental protocol. A calibration phase during which the subject moved freely and aimlessly provided data for the construction of the map *H* that transformed body movements into cursor movements. This was followed by an adjustment phase in order to match the control space to the computer screen. Training then started, divided in six blocks of nine repetitions of each of the six peripheral targets.

The subjects practiced the execution of reaching movements via an interface (Fig 6) that mapped an eight-dimensional signal space associated with upper body motions to the two-dimensional task space of a computer cursor. An array of four video cameras (V100, Naturalpoint Inc., OR, USA) was used to track active infrared light sources attached to the subject’s upper-body garments (two for each side of the body, one on the shoulder and one on the upper arm, as shown in Fig 6). Each camera pointed at a single marker, providing two signals defining the coordinates of the marker in the camera’s frame. Collectively, the four sensors provided an eight-dimensional body vector *q* that was transformed into a two-dimensional command vector *p* for controlling the position of a cursor on a computer screen, *p* = *Hq*. We show in S2 Fig the trajectories described by the each of the body sensors in the two-dimensional plane of its corresponding camera. Data is shown at different stages of learning, for each of the presented targets.

To design the matrix *H* for each subject, we used a standard linear dimensionality reduction method, principal component analysis (PCA) [62], to set up the dimensionality reduction implicit in the *q* to *p* map. PCA is based on the decorrelation of the input signals through the diagonalization of their covariance matrix; dimensionality reduction is obtained by keeping only the eigenvectors corresponding to the *K* largest eigenvalues. Here, these *K* = 2 eigenvectors became the rows of *H*. PCA provided us with a computationally straightforward method for identifying those directions that captured the largest range (i.e. the largest variance) of body motions within the space of sensor signals for each subject.

The interface map *H* from the input space of body signals to the output task space was constructed in three steps:

1. Calibration–Subjects were asked to freely explore their range of shoulders and upper arms motion in all possible directions for about 60 seconds. This stage results in an aimless free dance, during which subjects explored their available range of motion. The “calibration” data set *Q*_*CAL*_ was organized as an *S*x*M* matrix, where the number *S* of rows is the dimensionality of the space of sensor signals (here, *S* = 8) and the number *M* of columns is the total number of samples (here *M* = 5000, collected over about 66 seconds at 75 samples per second).
2. PCA–The principal components of the data set *Q*_*CAL*_ were extracted using PCA. The *S* eigenvectors of the covariance matrix of the sensor signals were ordered according to the magnitude of their corresponding eigenvalues, from largest to smallest. The eigenvalues represent the variance of the data along the eigenvector directions; those directions with high signal excursion correspond to larger eigenvalues. We considered those high-variance movement directions to be the user’s best controlled and most used combinations, and thus picked the *K* leading eigenvectors as rows to construct the *K*x*S* matrix *H* (Eq (5)). In our case the interface map *H* was a 2x8 matrix; the two first PCs accounted for 73±5% of the variance of the calibration dataset across subjects.
3. Mapping Adjustments–Although the two-dimensional subspace formed by the first two PCs captured a large fraction of the total variance of body motions, it did not necessarily reflect the natural up-down/left-right orientation of the display monitor. Therefore, following calibration and PC extraction, there was a customization phase in which users were allowed to set the origin, orientation, and scaling of the coordinates in task space, based on their preference.

After the calibration and mapping adjustments, subjects went through a two-hours training session. During training, the subjects used upper-body motions to control the movement of a cursor on a computer screen in order to reach a set of targets. Each trial started from the central target, which corresponded to the center of the screen; from there the subjects had to reach a peripheral target and return to center. The six peripheral targets were located 5 cm away from the center, equally spaced in directions 60° apart. The targets were presented randomly, with the condition that a given target was not presented until the subject had reached all other targets. The training session was organized into 6 blocks. Each block consisted of 9 sequences of reaches to the 6 targets, for a total of 54 center-out movements and the corresponding 54 returns; this amounts to 324 reaching movements for each session. The subjects had no visual feedback about the cursor position for the first 0.4 seconds following movement onset. The cursor then became visible, and the subjects could use visual feedback to correct for the reaching error if the cursor was not on target. During the initial 0.4 seconds without visual feedback, the subjects executed a ballistic movement based solely on their current estimate of the forward body-to-cursor map.

We show in S2 Fig the trajectories described by the cursor in the two-dimensional task space. Data is shown at different stages of learning, for each of the six presented targets. In addition, we show in S3 Fig, for all six subjects enrolled in the study, the time evolution during training of three indices that convey information about cursor movement: i) the time to reach the target, ii) the mean speed, and iii) the linearity index computed as the length of the trajectory from the central target to the external one normalized by the length of the straight line connecting these two targets.

### Data analysis

#### Estimation of the learning dynamics

To investigate the learning dynamics, we focused on the temporal evolution of two scalar variables in task space: the reaching error (RE) and the inverse model error (IME). These two variables were computed both from the data obtained from the subjects and from the synthetic data generated by simulating each subject-specific model of the individual learning process.

The reaching error RE was computed as the norm of the difference between the actual cursor position at the end of the reaching movement and the target position. For the experimental data, we considered as an estimate of the reaching error the distance between target and cursor at the end of the blind phase of the trial, which involved motions that relied only on the subject’s inverse internal model, in absence of visual feedback of the cursor motion. As the cursor reappeared, the subjects performed a corrective movement, bringing the cursor to target. The inverse model error IME was computed as the norm of the difference between the identity matrix *I*_*K*_ and the product *H G*^(*n*)^ between the actual interface map and the estimate of the inverse map at the end of each trial.

The lower dimensionality of the output space for the interface map *H* causes the problem of finding the inverse map to be ill-posed; the surface defined by the squared reaching error in the state space spanned by the components of *G* does not exhibit a single global minimum but a flat extended “valley” corresponding to all possible inverses of the interface map *H*. To circumvent this ambiguity and to monitor whether subjects converged to a stable inverse transformation, we estimated the inverse model matrix *G*^(*n*)^ from the subjects’ performance.

A typical experimental data set consisted of temporal sequences of reaching movements. At each trial *n* we considered a movement set, a sequence of *r* trials that included the *n*-th trial and the (*r*-1) trials that preceded it. Here we used *r* = 12, so that on average each movement set included two trials towards each of the six different targets. The body and target vectors for the *n*-th movement set were collected in the arrays

$$Q^{\left(n\right)}=[q^{\left(n-r+1\right)},\;\cdots ,q^{\left(n\right)}]\;and\;U^{\left(n\right)}=[u^{\left(n-r+1\right)},\;\cdots ,u^{\left(n\right)}]\;.$$ (16)

The matrix *G*^(*n*)^ then was obtained from a least-squares estimation based on *Q*^(*n*)^ = *G*^(*n*)^*U*^(*n*)^ (see Eq (3)):

$$G^{\left(n\right)}=Q^{\left(n\right)}\;U^{(n)T}{\left(U^{\left(n\right)}\;U^{(n)T}\right)}^{-1}\;.$$ (17)

The history of reaching errors for the *r* trials in the movement set that ended with trial *n* was computed as

$$E^{\left(n\right)}=HQ^{\left(n\right)}-U^{\left(n\right)}.$$ (18)

A scalar reaching error (RE) was then calculated by taking the spectral norm ∥*E*^(*n*)^∥ of the *K*x*r* matrix in Eq (18). Similarly, we calculated the inverse model error (IME) as the spectral norm ∥*H G*^(*n*)^ − *I*_*K*_∥. The IME is expected to approach 0 as learning converges and *G*^(*n*)^ approaches a right inverse of *H*. The convergence to a stable representation of the inverse map was assessed by computing the percentage difference in norm among consecutive estimations of *G*^(*n*)^,

$${\Delta G}^{\left(n\right)}=\frac{∥G^{\left(n\right)}-G^{\left(n-1\right)}∥}{∥G^{\left(n-1\right)}∥}.$$ (19)

We defined two additional errors to quantify whether each subject was also forming an estimate $\hat{H}$ of the forward map that converged to the interface map *H*. We defined the forward model error (FME) as

$${FME}^{\left(n\right)}=\frac{∥H-{\hat{H}}^{\left(n\right)}∥\;}{∥H∥}.$$ (20)

The current estimate ${\hat{H}\;}^{\left(n\right)}$ affects the prediction error, computed in task space as the difference between the actual position of the cursor and its estimated position based on the current estimate of the forward model. The L2 norm of this difference defines

$${PE}^{\left(n\right)}=∥p^{\left(n\right)}-{\hat{H}}^{\left(n\right)}\;q^{\left(n\right)}∥,$$ (21)

with *p*^(*n*)^ = *H q*^(*n*)^.

No moving window was used to compute these two errors, because both PE and FME could be extracted from the simulated data at each trial.

#### Model parameters

For each subject recruited for the study, we constructed a model that used the same interface map *H* as used by the subject and was exposed to the same target sequence. To set the individual learning rate *η* for the learning of the inverse map in Eqs (2) and (14), we fitted the experimentally observed decay of the norm RE of the reaching errors for each subject to an exponential of the form

$${RE}^{\left(n\right)}=ae^{-(\lambda_{RE})n\;}+c,$$ (22)

to obtain a value of *λ*_*RE*_ for each subject. We then set *η* = *λ*_*RE*_ in the corresponding subject-specific model.

To set values for the parameters *ε* and *σ* we adopted a minimum search approach to minimizing a cost function based on the forward model error (FME), as those two parameters mostly influence the evolution of the estimated forward model $\hat{H}$. The cost function *C* was defined as

$$C=\sum_{n=1}^{N}\frac{∥H-{\hat{H}}^{\left(n\right)}∥}{∥H∥},$$ (23)

where *N* is the total number of trials.

#### Comparison between simulated and real data

To estimate the similarity between real and model data we used the correlation coefficient R^2^. By definition, R^2^ evaluates similarity between the shapes of the compared curves and also provides additional information regarding the amplitude. This metric was applied to the norm RE of the reaching error, the norm IME of the inverse model error, the body coordinates *q*, and the cursor coordinates *p*. For each of these quantities, assume that the measured values are {*y*^(*n*)^}, 1 ≤ *n* ≤ *N*, where *N* is the total number of trials. For each one of these values, the model gives a prediction or estimation $\left\{{\hat{y}}^{\left(n\right)}\right\}$, 1 ≤ *n* ≤ *N*, with residuals $e^{\left(n\right)}=y^{\left(n\right)}-{\hat{y}}^{\left(n\right)}$, 1 ≤ *n* ≤ *N*. The mean of the observed data is given by $\overset{-}{y}=(1/N)\sum_{n=1}^{N}y^{\left(n\right)}$. The total sum of squares

$${SS}_{tot}=\sum_{n=1}^{N}{\left(y^{\left(n\right)}-\overset{-}{y}\right)}^{2}$$ (24)

is proportional to the variance of the experimentally observed values. The sum of squares of the residuals is

$${SS}_{res}=\sum_{n=1}^{N}{\left(y^{\left(n\right)}-{\hat{y}}^{\left(n\right)}\right)}^{2}=\sum_{n=1}^{N}{\left(e^{\left(n\right)}\right)}^{2}.$$ (25)

The most general definition of R^2^, as used here, follows from the ratio between these two sums of squares

$$R^{2}\equiv 1-\frac{{SS}_{res}}{{SS}_{tot}}.$$

## Supporting information

S1 Fig Modeling human learning as sequential learning of forward and inverse models. Data for the six subjects enrolled in the study (S1-S6). Model parameters in this sequential scenario were independently estimated for each subject as discussed in Methods. (a) Temporal evolution of the norm RE of the reaching error as a function of trial number ***n*** calculated from the experimental data (black) and from the model simulations (blue). (b) Temporal evolution of the norm IME of the inverse model error, the difference between the identity matrix ***I*_*K*_** and the product of the interface map *H* and the inverse model ***G***^(***n***)^, estimated from the experimental data (black) and from the model simulations (blue). Both metrics were calculated over a moving window that includes the current and its 11 preceding trials.
(TIF)

S2 Fig Example of body and cursor data. Body signals and cursor trajectories across training, from a representative subject. Body signals during each reach are shown as the motions of the four body markers in the frame of the corresponding cameras; camera labels and scales in pixels along camera axes are shown only for the top left panel. All trajectories are color coded to identify target direction, and shown for the 400 ms following movement onset; the cursor was not visible to the user during this period. Scale for cursor motions is also shown only for the top left panel. Cursor and body-signal trajectories are shown for the first and last set of reaches for block 1 (top panels) and block 6 (bottom panels).
(TIF)

S3 Fig Cursor control in the task space. Data for the six subjects enrolled in the study (S1-S6). Temporal evolution of the (a) time to reach the target, (b) mean speed, and (c) linearity index, defined as the length of the trajectory from the central target to the peripheral target divided by the length of the straight line connecting the two. All three indices were calculated over a moving window that includes the current and its 11 preceding trials.
(TIF)
