# Supplementary figures and images for: The dynamics of motor learning through the formation of internal models
Source: PLoS Comput Biol. 2019 Dec 20;15(12):e1007118. doi: 10.1371/journal.pcbi.1007118 (PMC6944380; doi:10.1371/journal.pcbi.1007118)

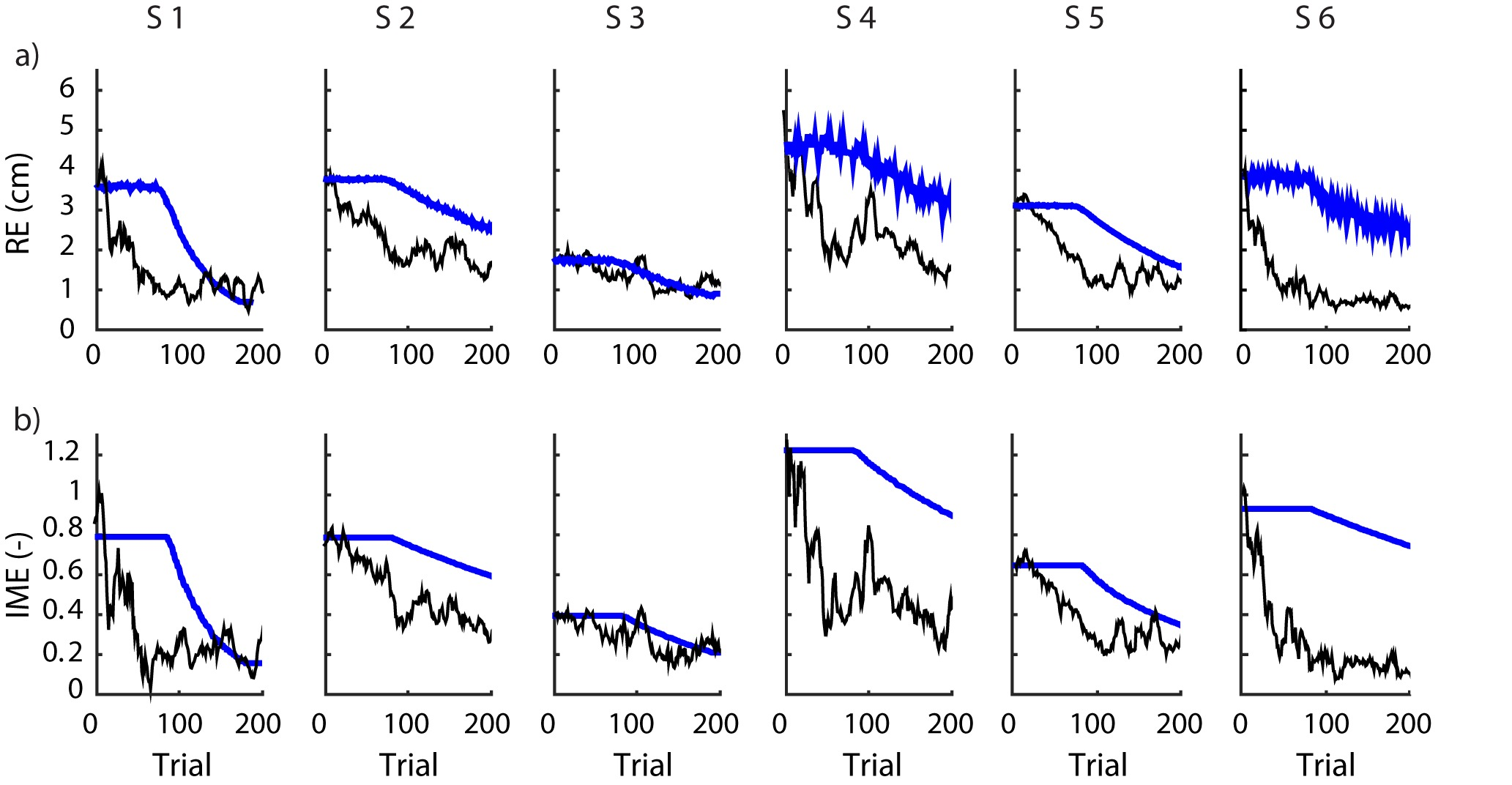

Supplement: S1 Fig — Data for the six subjects enrolled in the study (S1-S6). Model parameters in this sequential scenario were independently estimated for each subject as discussed in Methods. (a) Temporal evolution of the norm RE of the reaching error as a function of trial number n calculated from the experimental data (black) and from the model simulations (blue). (b) Temporal evolution of the norm IME of the inverse model error, the difference between the identity matrix IK and the product of the interface map H and the inverse model G(n), estimated from the experimental data (black) and from the model simulations (blue). Both metrics were calculated over a moving window that includes the current and its 11 preceding trials. (TIF) [file pcbi.1007118.s001.tif]

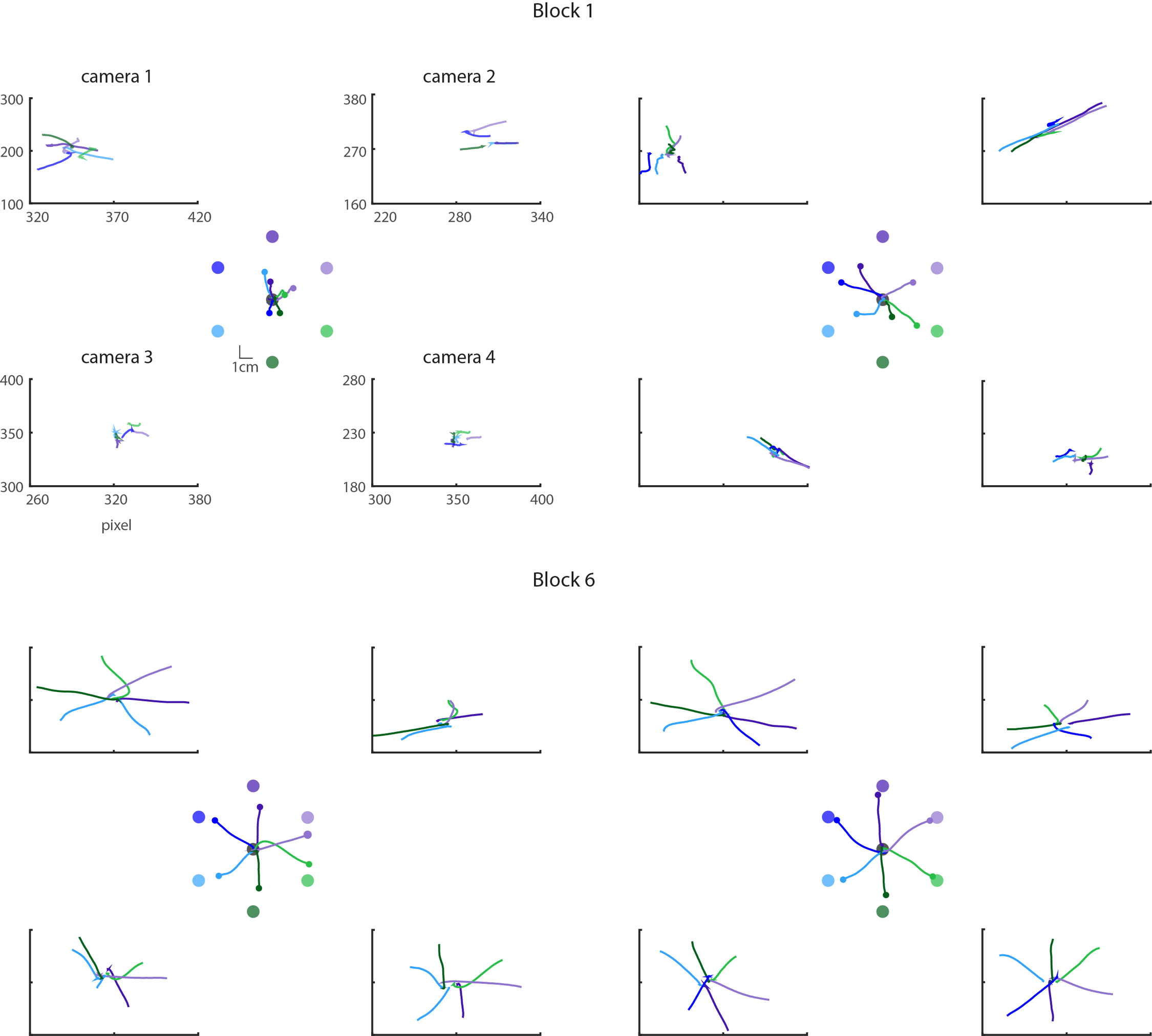

Supplement: S2 Fig — Body signals and cursor trajectories across training, from a representative subject. Body signals during each reach are shown as the motions of the four body markers in the frame of the corresponding cameras; camera labels and scales in pixels along camera axes are shown only for the top left panel. All trajectories are color coded to identify target direction, and shown for the 400 ms following movement onset; the cursor was not visible to the user during this period. Scale for cursor motions is also shown only for the top left panel. Cursor and body-signal trajectories are shown for the first and last set of reaches for block 1 (top panels) and block 6 (bottom panels). (TIF) [file pcbi.1007118.s002.tif]

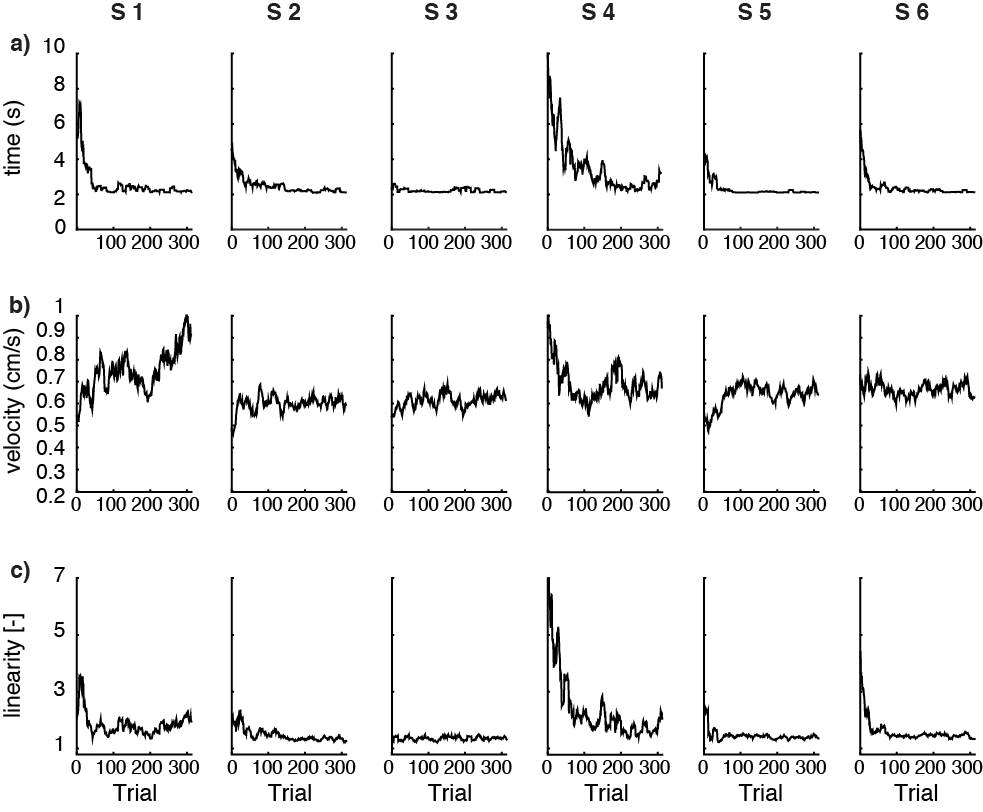

Supplement: S3 Fig — Data for the six subjects enrolled in the study (S1-S6). Temporal evolution of the (a) time to reach the target, (b) mean speed, and (c) linearity index, defined as the length of the trajectory from the central target to the peripheral target divided by the length of the straight line connecting the two. All three indices were calculated over a moving window that includes the current and its 11 preceding trials. (TIF) [file pcbi.1007118.s003.tif]
